# Person-centred online lifestyle coaching in childhood, adolescent, and young adult cancer survivors: protocol of the multicentre PanCareFollowUp lifestyle intervention feasibility study

**DOI:** 10.1186/s40814-022-01221-x

**Published:** 2022-12-16

**Authors:** Eline Bouwman, Rosella P. M. G. Hermens, Morven C. Brown, Vera Araújo-Soares, Nicole M. A. Blijlevens, Tomas Kepak, Katerina Kepakova, Leontien C. M. Kremer, Selina R. van den Oever, Helena J. H. van der Pal, Roderick Skinner, Saskia M. F. Pluijm, Jacqueline J. Loonen, on behalf of the PanCareFollowUp Consortium, Renée L. Mulder, Rebecca J. van Kalsbeek, Lars Hjorth, Cecilia Follin, Lill Eriksson, Thomas Relander, Jacob Engellau, Karin Fjordén, Karolina Bogefors, Anna S. Holmqvist, Riccardo Haupt, Monica Muraca, Brigitte Nicolas, Francesca Bagnasco, Marina Benvenuto, Anna Aulicino, Luca Laudisi, Hana Hrstkova, Viera Bajciova, Marta Holikova, Lucie Strublova, Anne Uyttebroeck, Marleen Renard, Sandra Jacobs, Heidi Segers, Monique van Helvoirt, Jeanette F. Winther, Luzius Mader, Line E. Frederiksen, Elisabeth A. W. Andersen, Gisela Michel, Stefan Boes, Katharina Roser, Irene Göttgens, Iridi Stollman, Adriaan Penson, Dionne Breij, Vera Araujo-Soares, Samira Essiaf, Anne Blondeel, William Sciberras, Joke Korevaar, Mieke Rijken, Anita Kienesberger, Jaap den Hartogh, Hannah Gsell, Carina Schneider, Jaap den Hartogh, Edit Bardi, Jeroen te Dorsthorst

**Affiliations:** 1 Centre of Expertise for Cancer Survivorship, Radboud Institute for Health Sciences, Radboud University Medical Centre, Reinier Postlaan 4, 6500 HB Nijmegen, the Netherlands; 2 Scientific Institute for Quality of Healthcare (IQ Healthcare), Radboud Institute for Health Sciences, Radboud University Medical Centre, Geert Grooteplein Zuid 10, 6525 GA Nijmegen, the Netherlands; 3 Wolfson Childhood Cancer Research Centre, Newcastle University Centre for Cancer, Newcastle University, Herschel Building, Brewery Lane, Newcastle upon Tyne, NE1 7RU UK; 4 Population Health Sciences Institute, Newcastle University, Ridley Building 1, Queen Victoria Road, Newcastle upon Tyne, NE1 7RU4 UK; 5 Department of Health Technology & Services Research, Technical Medical Centre, University of Twente, P.O. Box 217, 7500 AE Enschede, the Netherlands; 6 International Clinical Research Centre (FNUSA-ICRC), St. Anne’s University Hospital, Masaryk University, Pekařská 53, 656 91 Brno, Czech Republic; 7 Princess Máxima Centre for Paediatric Oncology, Heidelberglaan 25, 3584 CS Utrecht, the Netherlands; 8 Department of Paediatrics, Emma Children’s Hospital, Amsterdam UMC, Meibergdreef 9, 1105 AZ Amsterdam, the Netherlands; 9 Faculty of Medicine, Utrecht University and Utrecht Medical Centre, Universiteitsweg 98, 3584 CG Utrecht, the Netherlands; 10 PanCare, Jacobus Bellamylaan 16, 1401 AZ Bussum, the Netherlands; 11 Great North Children’s Hospital, Royal Victoria Infirmary, Queen Victoria Road, Newcastle upon Tyne, NE1 4LP UK; 12 Translational and Clinical Research Institute, Wolfson Childhood Cancer Research Centre, Herschel Building, Brewery Lane, Newcastle upon Tyne, NE1 7RU UK; 13 Department of Haematology, Radboud University Medical Centre, Radboud Institute for Health Sciences, Geert Grooteplein Zuid 10 , 6525 GA Nijmegen, Nederland

**Keywords:** Childhood, adolescent, and young adult cancer survivors, eHealth, Screen-to-screen, Lifestyle, Physical activity, Diet, Person-centred care, Motivational interviewing, Coaching, Feasibility

## Abstract

**Background:**

Physical inactivity and unhealthy dietary habits are known to be disadvantageous for the development of late adverse effects in survivors of childhood, adolescent, and young adult cancer. To make interventions, aimed at improving lifestyle, fit into the daily life of survivors, interventions should be designed and delivered in a person-centred way with a limited time burden. As part of the European PanCareFollowUp project, an eHealth intervention was developed to support sustainable changes to physical activity levels and/or diet of childhood, adolescent, and young adult cancer survivors. This feasibility study aims to gain insight into the feasibility and potential effect sizes of the PanCareFollowUp lifestyle intervention.

**Methods:**

The PanCareFollowUp lifestyle intervention consists of person-centred 3–6 screen-to-screen sessions with a certified lifestyle coach. The intervention will be evaluated with a single-arm pre-post feasibility study conducted at two survivorship care clinics in the Netherlands. A total of 60 participants who are (i) diagnosed with cancer <25 years, (ii) ≥ 5 years post-treatment, (iii) aged 16–55 years, and (iv) have a low physical activity level and/or unhealthy dietary intake manifested by overweight will be recruited. Using reports, hospital records, and questionnaires for survivors, coaches, and late effect doctors, feasibility will be based on (i) adherence to intervention, (ii) acceptability, (iii) practicality, (iv) integration/implementation, (v) demand, and (vi) attrition. The potential effect sizes of the intervention will be explored by determining the percentage of survivors that reach the personalized lifestyle goals that were set with the coach. Physical activity level, dietary intake, BMI, general self-efficacy, self-management, and motivation level will be assessed at three time points with questionnaires, reports, and/or an accelerometer.

**Discussion:**

Data of this study will be gathered to assess the feasibility and potential effect sizes. This will allow for further intervention refinement as needed as well as to inform a future large-scale intervention study and a manual for implementation at other centres.

**Trial registration:**

International Clinical Trial Registry Platform (ICTRP) number: NL8932 (ICTRP Search Portal (who.int)). Registered on September 29, 2020.

**Supplementary Information:**

The online version contains supplementary material available at 10.1186/s40814-022-01221-x.

## Introduction

Children diagnosed with cancer now have a far better prognosis with a 5-year survival rate of over 80% compared to 30% in the 1970s [1–3]. A similar, though slightly less dramatic, improvement in successful cancer cures can be observed in survivors of adolescent and young adult cancer [4, 5]. Therefore, the childhood, adolescent, and young adult (CAYA) cancer survivor population is rapidly increasing. Currently, there are over 500,000 CAYA cancer survivors living in Europe and this number grows by approximately 12,000 CAYA cancer survivors each year [6]. However, due to their cancer treatment, CAYA cancer survivors are at high risk for developing chronic health and psychosocial problems, also known as late effects. These late effects can be severe and often result in excess morbidity and mortality compared to peers of the same age in the general population [7–10].

An unhealthy lifestyle, for instance characterized by a low physical activity level and unhealthy dietary habits, increases the already heightened risk of developing late effects in CAYA cancer survivors [11–13]. The beneficial effects of complying with healthy behaviours are illustrated in a study by Jones et al. in which it was shown that adherence to national vigorous exercise guidelines (i.e. ≥9 MET hours/week) in Hodgkin lymphoma survivors was associated with a 51% lower risk of any cardiovascular event compared with Hodgkin lymphoma survivors not meeting the guidelines [11]. Likewise, in a study by Schindera et al., higher aerobic fitness has been associated with a lower chance of having cardiovascular risk factors, such as having a high waist circumference, low HDL cholesterol levels, high triglyceride levels, a high composite cardiovascular risk score, and metabolic syndrome in childhood cancer survivors (CCSs) [14]. Previous research also showed that among survivors of childhood leukaemia, a Mediterranean diet, including a high intake of fruit and vegetable and a low intake of meat, has been negatively associated with having the metabolic syndrome, meaning that for each point higher on the Mediterranean Diet Score (range 0–8 with 8 representing full adherence to the Mediterranean diet), the odds of having the metabolic syndrome declined by 31% [15]. For this reason, cancer organizations and cancer survivorship guidelines recommend cancer survivors to participate in regular physical activity and maintain a healthy diet [16].

However, despite these evidence-based recommendations, CAYA cancer survivors often fail to meet them. Several studies have reported that intakes of fibre, fruit, and vegetables are often inadequate in survivors [17–19], whereas other studies report a high proportion of survivors not meeting the physical activity recommendations [19, 20]. Low compliance of CAYA cancer survivors to the recommendations may be explained by potential physical (e.g. limb amputation, heart failure) and psychological (e.g. fatigue, depression) late effects due to their cancer history and/or treatment regimes. Therefore, being physically active and/or complying with a healthy diet can be challenging for CAYA survivors [21]. Proper guidance in the form of interventions given by healthcare professionals with knowledge of late effects and related limitations can be helpful for CAYA cancer survivors to achieve healthy behaviours.

Delivering interventions according to the concept of person-centred care seems a promising approach for the heterogeneous population of CAYA cancer survivors [22, 23]. Person-centred care enables a partnership between the patient and their healthcare professional and is thought to be an important contributing factor in stimulating self-efficacy and self-management [24]. In addition, it ensures that care meets the physical, mental, and social health needs of each individual survivor.

Moreover, in order to make the interventions fit into the daily lives of CAYA cancer survivors, lifestyle interventions should be delivered in a way that limits time and travel burden as much as possible. For this reason, electronic health (eHealth) may be an attractive mode of lifestyle intervention delivery in this population as it avoids unnecessary clinic visits and limits impact on working life and care duties for CAYA cancer survivors. Though these interventions have not been extensively tested in CAYA cancer survivors, Beleigoli et al. report in their systematic review that web-based digital interventions led to greater short-term weight loss than offline interventions in overweight and obese adults [25]. In addition, in a study by Costello et al. on the delivery of survivorship care via telemedicine, all participating childhood cancer survivors would recommend telemedicine to family and friends and 94.1% would choose to have another telemedicine visit in the future [26].

As part of the European Union Horizon 2020-funded PanCareFollowUp (PCFU) project, the PCFU lifestyle intervention was developed to support the adoption of positive and sustainable changes in lifestyle behaviours (i.e. diet and/or physical activity) in CAYA cancer survivors [27]. The primary aim of the PCFU lifestyle intervention feasibility study is to gain insight into the intervention’s feasibility in terms of adherence to the intervention, acceptability, practicality, integration, implementation, demand, and attrition. The second aim is to assess potential effect sizes regarding CAYA cancer survivors reaching their personalized goal(s). These goals are set together with a lifestyle coach to adapt and sustain their desired lifestyle change behaviour. Lastly, short- and long-term changes in terms of physical activity level, dietary intake, body mass index (BMI), general self-efficacy, self-management, and motivation level will be assessed at three time points.

## Methods

### Design and setting

The PCFU lifestyle intervention study—a prospective single-arm pre-post feasibility study with three measurement moments (T0 at baseline, T1 after the last session, and T2 at 4 months follow-up)—will be conducted at two survivorship clinics in the Netherlands: one situated at a university medical centre and one situated at a specialized clinic for childhood cancer. After recruitment of survivors at the clinic, the PCFU lifestyle intervention will be delivered remotely through screen-to-screen video calling with a lifestyle coach. The coach will provide the intervention from a computer at his/her workplace at one of the participating clinics or own practice. This study protocol will be reported in accordance with the SPIRIT guidelines (see Additional file 1) [28] and TIDieR checklist (see Additional file 2) [29].

### Study participants

In total, 60 participants (30 per centre) will be recruited for participation in this feasibility study (see the “Sample size” section). Eligible participants must meet all of the following inclusion criteria at study time: (1) is a survivor of childhood, adolescent, or young adult (CAYA) cancer (diagnosed with any type of cancer under the age of 25 years to include most survivors attending the survivorship care clinics); (2) is at least 5 years from the end of cancer treatment given that after this period survivors make the transition from the oncologists to a long-term follow-up at survivorship care clinics; (3) cancer free at time of the study; (4) is aged 16–55 years at time of inclusion given that this age group is most likely to be independent regarding dietary patterns as well as acquainted with video calling software; (5) not meeting the World Health Organization (WHO) norm for physical activity (exercising ≥150 min of moderate-intensity aerobic physical activity per week or ≥75 min of vigorous-intensity aerobic physical activity per week or an equivalent combination of moderate- and vigorous-intensity activity, and should also do muscle-strengthening activities at moderate or greater intensity that involve all major muscle groups on 2 or more days a week) and/or has an unhealthy dietary intake manifested by overweight (BMI ≥ 25 kg/m^2^) (see Table 1); and (6) is motivated to change unhealthy lifestyle behaviour(s) according to the criteria of the RICk (readiness, importance, confidence, and knowledge) criteria [30] as assessed by the late effect doctor. Excluded are survivors who meet at least one of the following criteria: (1) diagnosed with Down syndrome, (2) diagnosed with cognitive disorders, (3) has depressive symptoms as assessed with anamnesis by the late effect doctors and/or by the Hospital Anxiety and Depression Scale (anxiety and/or depression score ≥8), (4) is diagnosed with complex endocrine disorders that might influence weight or other conditions that limit the survivor’s ability to engage in health promotion discussions and activities, (5) is already participating in an intervention study or other interventions aiming to improve lifestyle behaviours, (6) is underweight (BMI ≤ 20 kg/m^2^), or (7) has severe physical limitations which will hinder proper participation in the intervention as assessed by the late effect doctor. Lastly, to assess the feasibility of the PCFU lifestyle intervention, the lifestyle coaches and late effect doctors will be asked to complete several questionnaires as well.

**Table 1:** Questionnaire to assess physical activity levels according to the WHO norms for adults aged 18–64

1. During the past month, did you participate in any physical activities or exercises such as walking for exercise, running, tennis, soccer, volleyball, golf, gardening, bicycling, swimming, wheelchair basketball, weight training, yoga, resistance exercises, or jumping rope? 1.1 In case yes, now thinking about the vigorous physical activities you do in a usual week, do you do vigorous activities for at least 10 min at a time, such as running, aerobics, tennis, soccer, volleyball, wheelchair basketball, heavy yard work, or anything else that causes large increases in breathing or heart rate? 1.1.1. In case yes, how many days per week do you do these vigorous activities for at least 10 min at a time? …………………………………….. days per week 1.1.2. In case yes, on days when you do vigorous activities for at least 10 min at a time, how much total time per day do you spend doing these activities on average? ……………… minutes per day on average 1.2. Now thinking about the moderate physical activities you do in a usual week, do you do moderate activities for at least 10 min at a time, such as brisk walking, bicycling, vacuuming, gardening, manual operation of a wheelchair, or anything else that causes small increases in breathing or heart rate? 1.2.1. How many days per week do you do these moderate activities for at least 10 min at a time? …………………………………….. days per week 1.2.2. On days when you do moderate activities for at least 10 min at a time, how much total time per day do you spend doing these activities on average? ……………… minutes per day on average 1.3. Now thinking about the muscle-strengthening activities such as weight training, push-ups, sit-ups, yoga, resistance exercises, or jumping rope, do you do these kinds of muscle-strengthening activities in a usual week? 1.3.1. How much total time per week do you spend doing these activities on average? …………… minutes per week on average	

Participant recruitment will take place at two survivorship care clinics. On arrival at the survivorship care clinic for a routine follow-up visit, a healthcare professional (e.g. a nurse or physician assistant) will perform a weight and height measurement of the survivor to determine the survivor’s BMI as part of routine clinical care. The late effect doctor and/or nurse practitioner will be provided with this information prior to the start of the consultation which will serve as a conversational opener to discuss the survivor’s lifestyle behaviours, including dietary intake and physical activity level. As part of screening procedures of the study, the late effect doctor/nurse practitioner will assess the survivor’s compliance with the WHO norm for physical activity using a short questionnaire based on the Global Physical Activity Questionnaire (see Table 1) [31–33]. Depending on the survivorship care clinic, the questionnaire will either be completed together, or the survivor will complete the questionnaire by himself before the consultation. All survivors will be verbally informed by the late effect doctor about the importance of a healthy lifestyle to prevent late health effects or improve their health outcomes. Subsequently, the doctor will check if the survivor meets the eligibility criteria of the PCFU lifestyle intervention. If applicable, the doctor will assess, based on the narrative of the survivor, the motivation and willingness of the survivor to change his/her health behaviour(s). Therefore, to assess qualification for the intervention, the late effect doctor/nurse practitioner uses the following checkpoints according to the RICk criteria: survivor’s readiness to change his/her lifestyle, the survivor knows the importance of changing his/her lifestyle, and the survivor has confidence that he/she will be able to succeed in changing his/her lifestyle and has the knowledge he/she needs before participating in a lifestyle intervention [30]. As the intervention aims to establish sustainable lifestyle changes in survivors, it is important that survivors are ready to commit to lifestyle changes.

The late effect doctor will register whether the survivor is motivated according to the RICk criteria on the registration form. In case of a survivor fulfilling the inclusion and exclusion criteria, he/she will be introduced to the intervention and study and given brief information verbally. If the survivor shows interest in participation, he/she is provided with an information package containing an information letter and an informed consent form. Within 2 weeks after the clinic visit, the survivor will be contacted by phone by the local investigator/research nurse. During this phone call, the survivor has the opportunity to ask questions. If the survivor decides to participate in the PCFU lifestyle intervention study, he/she will be instructed to sign the informed consent form and return it to their treating survivorship care clinic using a postage paid envelope. When informed consent is received by the study team, the survivor will be registered for the intervention. Subsequently, to ensure participant confidentiality, the survivor will be assigned an identification code for all data collected in the study as well as for data on sociodemographic characteristics, cancer history, and treatment.

### PanCareFollowUp lifestyle intervention

#### Development

To develop the PCFU lifestyle intervention, an approach guided by the 2008 Medical Research Council Framework for the development and evaluation of complex interventions was used [34]. Furthermore, a systematic review, including two research aims, and two qualitative studies were performed to create an adequate evidence base. The first aim of the systematic review focused on facilitators and barriers of adopting a healthy lifestyle in CAYA cancer survivors (i.e. regular physical activity, a healthy diet, a healthy body weight, no smoking, limited alcohol consumption, no drug use, good sleep habits, and limited and protected sun exposure), whereas the second review focused on the effectiveness and the effective components of eHealth lifestyle interventions developed for CAYA cancer survivors. To gain further insight into the barriers and facilitators of providing health behaviour support to CAYA cancer survivors, we conducted focus groups with healthcare professionals from survivorship care clinics across Europe. Lastly, the other qualitative study aimed to assess the facilitators and barriers of adopting a healthy lifestyle as perceived childhood cancer survivors with a Dutch or another European nationality.

In addition, the protocol of nurse-led video-coaching (REVIVER) care interventions, as developed by late effect doctors and nurses at the Radboud University Medical Centre (Nijmegen, the Netherlands), served as a starting point for the development of the PCFU lifestyle intervention [35]. The REVIVER interventions involve coaching sessions with CAYA cancer survivors delivered by trained nurses via screen-to-screen video calling. The interventions aim to either reduce symptoms of cancer-related fatigue, stimulate self-efficacy, or change lifestyle behaviours in CAYA cancer survivors.

In order to develop the new PCFU lifestyle intervention, the REVIVER lifestyle intervention has been refined using input from experiences of the REVIVER interventions as well as the results of the systematic review and qualitative studies. Adaptations have been made on the structure of the intake session, coaching sessions, and reflection session in terms of items discussed during the sessions and planning of the sessions. In addition, contextual factors such as training of the coaches have been adjusted. Lastly, the intervention will be evaluated on other outcomes with other evaluation measures compared with the REVIVER intervention to capture the potential effect sizes and feasibility of the PCFU lifestyle intervention.

#### Concept

The goal of the PCFU lifestyle intervention is to promote the adoption of sustainable changes in lifestyle behaviour in CAYA cancer survivors with the long-term goal of preventing cardiovascular, pulmonary, and musculoskeletal diseases. The intervention targets CAYA cancer survivors with a low physical activity level and/or an unhealthy dietary intake manifested by overweight (BMI ≥25). The PCFU lifestyle intervention elaborates on the concepts of person-centred care which aims to empower survivors to take charge of their health. In addition, the intervention applies multiple behaviour change techniques (see Additional file 1) and involves synchronous participation by survivors and eHealth lifestyle coaches.

#### Delivery

The PCFU lifestyle intervention is individually and remotely delivered through a secure screen-to-screen video calling software for online meetings. The application and use of the video calling software (Zaurus® or Skype for Business®) is free of charge and adheres to the General Data Protection Regulation. Therefore, the survivor needs to be in the possession of a smartphone, tablet, or computer. The survivor can participate either at home or at another desired location with a secure and stable internet connection. During the intervention session, the lifestyle coach makes use of two screens, which enables the coach to share one screen with relevant information with the CAYA cancer survivor (e.g. websites with information on physical activity and/or dietary intake).

The intervention will be delivered by lifestyle coaches affiliated with one of the two centres and in the possession of certificates for lifestyle coaching. Both coaches are trained in person-centred care by a person-centred care expert of the Radboud University Medical Centre (Nijmegen, the Netherlands). This centre has been certified by Planetree International for excellence in person-centred care. In addition, both coaches are trained for the intervention and qualified for applying motivational interviewing. Lastly, as CAYA cancer survivors are a unique population with specific needs, the coaches are either trained in late effects by doctors with expertise in this field or had already years of working experience in the field of survivorship care.

#### Content and structure

To support CAYA cancer survivors in adopting a healthier lifestyle, multiple behaviour change techniques are applied by the coach in the PCFU lifestyle intervention. These behaviour change techniques with their presumed mechanisms of action are described in the table in S1 Table [36, 37]. In addition, the intervention coach uses principles of person-centred care (i.e. listening to the wishes and preferences of the CAYA cancer survivor) to establish a partnership between the CAYA cancer survivor and the coach.

The intervention consists of an intake session and 3 to 6 screen-to-screen coaching sessions delivered within a period of 3–4 months. Approximately every 2–3 weeks, a session will be scheduled according to the preferences of the survivor. The exact number, frequency, and duration of the coaching sessions depend on the personal preferences of the participating survivor. The average duration of a session will be approximately 30–45 min.

The goal of the intake session is to reach a consensus on which unhealthy lifestyle behaviour(s) will be the main focus for the upcoming months. Together with the lifestyle coach, the participating survivor will set a personal goal that he/she would like to reach by the last coaching session. This goal can either be focused on increasing physical activity and/or adopting healthier dietary habits. Considering personal needs and preferences, the coach and survivor together decide on the best plan of action. This also includes setting smaller, short-term goals. Lastly, potential barriers and facilitators for reaching the personal health goal are discussed. Subsequently, during the regular coaching sessions that follow, the coach and the survivor will go through the following phases in motivational interviewing: (i) engaging with the survivor, (ii) focusing on target behaviour and goal setting, (iii) evoking by drawing out the survivor’s intrinsic motivation to change, and (iv) planning to make clear goals and plans to change behaviours. Each session starts with exploring the survivor’s Stages of Change [38], a reflection on the past time, and whether the short-term goals—and overall goal—have been reached yet. Next, the coach and survivor discuss plans to overcome lifestyle behaviour change barriers experienced during the previous weeks (if applicable). If needed, the coach and survivor will, in mutual agreement, adjust the plan of action for the survivor to reach the overall health goal. During the last (regular) coaching session, the lifestyle coach and survivor reflect on the intervention, the progress made, and whether the overall goal has been reached. In addition, a final reflection session will be scheduled 4 months ahead. Until then, the survivor is tasked to sustain the lifestyle behavioural changes made by him-/herself. During the reflection session, the survivor’s progress will again be reviewed and the coach and survivor discuss strategies on how to make sure the lifestyle goals reached are sustainable.

#### Fidelity

The fidelity of the intervention will be pursued by evaluating the self-reported adherence of the coach to the intervention protocol. Following every intake, coaching, or reflection session, the coach needs to fill in an online report including questions on topics that should be covered during the session. This will serve as a prompt to discuss all these items in the sessions. Secondly, fidelity in terms of adherence of coaches to the intervention protocol will also be reviewed with the PCFU lifestyle intervention evaluation questionnaire of CAYA cancer survivors. In this way, it can be checked whether all intended items are discussed. Thirdly, fidelity can be assured as the involved coaches are trained in person-centred care, motivational interviewing, lifestyle coaching, and the intervention protocol. Lastly, results by coaches of both participating survivorship care centres will also be displayed per centre in the main paper to compare results as a means to check for fidelity.

### Data collection and outcomes

**Fig. 1:**
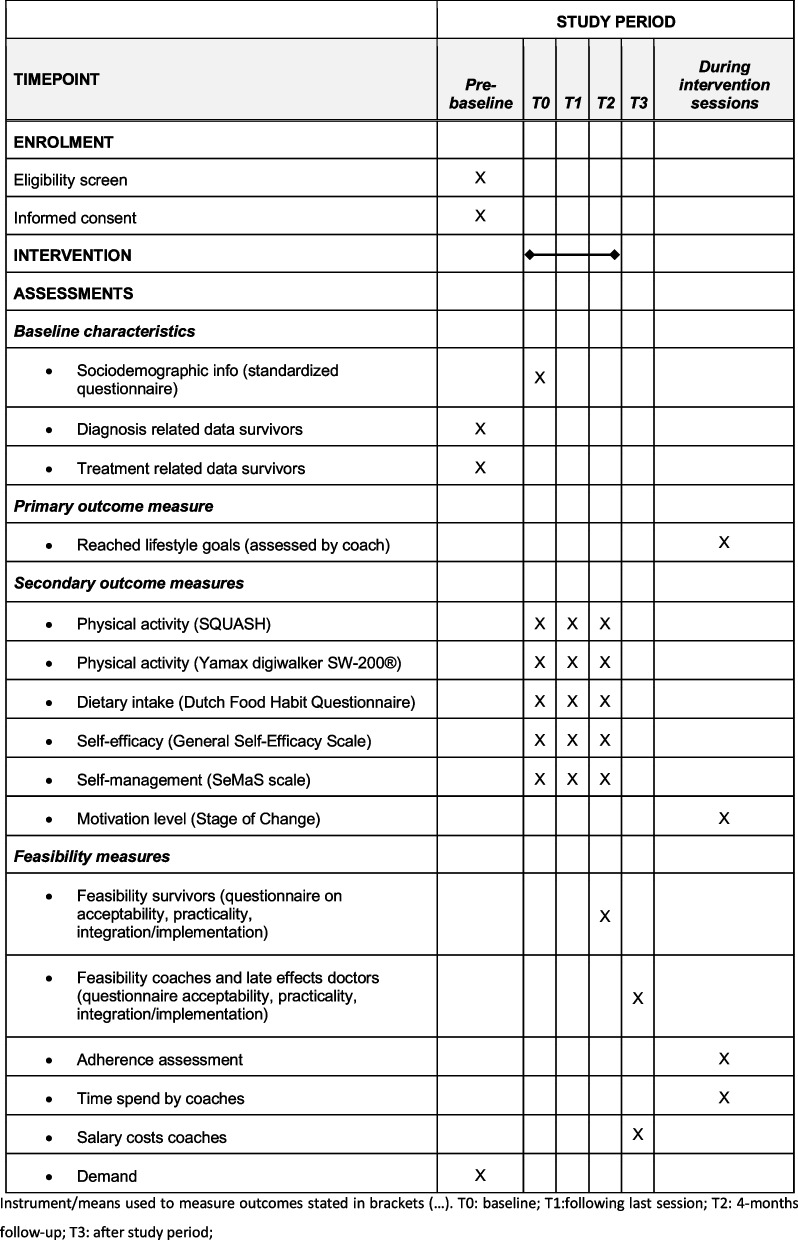
Schedule of enrolment, intervention, and assessments

**Fig. 2:**
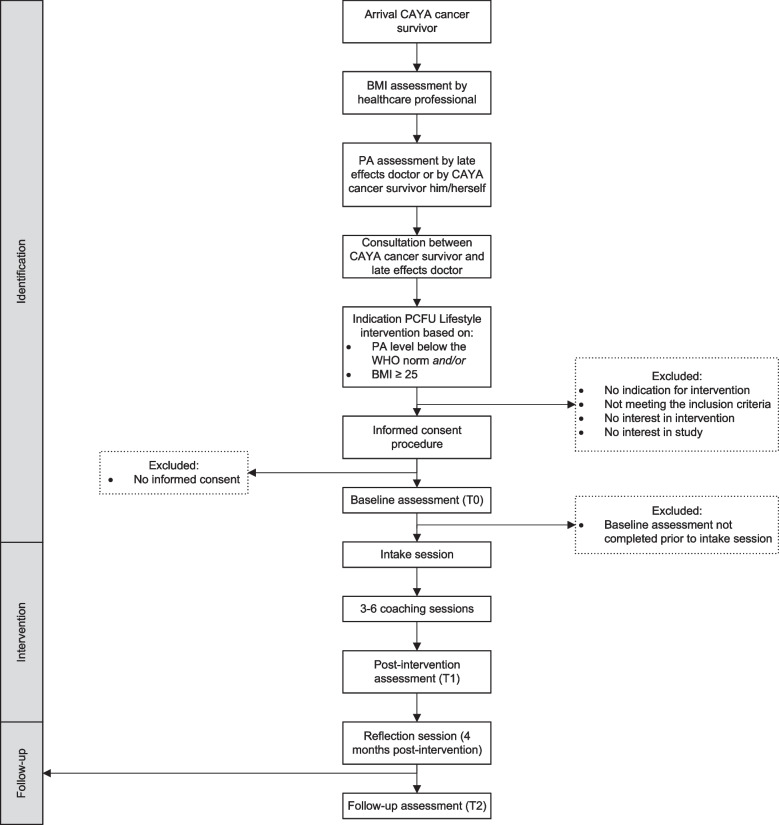
Flowchart of participant recruitment and involvement in the PanCareFollowUp lifestyle intervention study

The schedule of enrolment, the intervention, and assessments performed with the PCFU lifestyle intervention study are displayed in Fig. 1. The study flow of the PCFU lifestyle intervention study is detailed in Fig. 2. All data will be stored in an electronic data capture system (Castor EDC) compliant with all relevant regulations regarding research with servers located in the Netherlands. Data from participating CAYA cancer survivors on cancer diagnosis and treatment are entered into the database prior to the start of the intervention (pre-baseline). For CAYA cancer survivors, the measurements will take place before the intake session at baseline (T0), immediately following the last session with the coach (T1), and 4 months after the last session on completion of the reflection session (T2). For the lifestyle coaches and referring late effect doctors, one measurement will take place at the end of the study period (T3).

#### Baseline characteristics

With the CAYA survivor’s consent, cancer- and treatment-related data such as primary cancer diagnosis, age at diagnosis, and chemo- or radiotherapies will be retrieved from the medical records and entered in the study database. Sociodemographic information of the participants will be assessed with a standardized questionnaire including questions on age, educational level, employment status, and country of birth. The educational level will be scored in the categories “low”, “middle”, and “high” according to the Dutch standard education classification (27). Employment status will be categorized as either “unemployed”, “employed”, or “being a student”.

#### Feasibility evaluation

Feasibility of the intervention as a primary outcome measure will be assessed on the following indices: adherence, acceptability, practicality, integration/implementation, demand, and attrition [39].

##### Adherence

Adherence of CAYA cancer survivors to the PCFU lifestyle intervention will be measured by comparing the scheduled appointments for the intervention sessions with the actual attended appointments with survivors. This will be reported in the online database by the coach after each intervention session with a survivor. Coaches’ reports of the coaching session will be assessed to evaluate the adherence to the PCFU lifestyle intervention. The coach will record all made appointments for sessions, whether the survivors complied with the sessions.

##### Acceptability

Acceptability of the PCFU lifestyle intervention will be self-assessed with questionnaires (developed by the research team) which include questions on satisfaction with the content and delivery of the intervention. This questionnaire will be provided to the participating CAYA cancer survivors on completion of the reflection session (T2). The lifestyle coaches and referring late effect doctors will receive a questionnaire on acceptability at the end of the study period (T3). Questions for the healthcare professionals focus on the general view on the referral process (for late effect doctors only), content, duration, and amount of sessions.

##### Practicality

Practicality of the PCFU lifestyle intervention in terms of intervention delivery will be measured by a self-assessed questionnaire developed by the research team. This questionnaire is provided to CAYA cancer survivors at T2 and the lifestyle coaches and late effect doctors at T3. In addition, practicality in terms of costs of the intervention will be evaluated by applying a micro-costing approach that seeks to identify the costs of all underlying activities of the intervention. Therefore, time spent by coaches for tasks related to the PCFU lifestyle intervention will be collected directly from the coaches’ reports. For every consultation, an estimate of the time needed to prepare a session, execute, and document a session will be collected. In addition, hospital records will be utilized to retrieve the salary costs of the lifestyle coaches to calculate the costs of the intervention.

##### Integration/implementation

A self-assessed questionnaire will be used to assess the successfulness of the integration and implementation of the PCFU lifestyle intervention. In this questionnaire (developed by the research team), questions on facilitators and barriers and successes and failures of the intervention are included. This questionnaire will be provided to the CAYA cancer survivors (at T2). The lifestyle coaches and late effect doctors will be provided with this questionnaire at T3. In addition, after every session, potential facilitators and barriers experienced by the survivor with the intervention are reported by the coaches.

##### Demand

To assess the demand for the intervention, all potential eligible survivors as well as their expressed interest in the intervention will be listed using a self-compiled registration form. Late effect doctors involved in referrals to the PCFU lifestyle intervention study will be instructed to fill in this form during consultations with survivors. It contains questions on whether the survivor complies to the eligibility criteria, whether the survivor is motivated to change his/her health behaviour(s), and whether he/she is interested in the PCFU lifestyle intervention study. The rate of interested survivors compared to those eligible survivors can be calculated from this registration form.

##### Attrition

Data will be collected on measurements (i.e. questionnaires and accelerometers) completed by participating survivors at all measurement points (T0, T1, and T2). In addition, data on when measurements are completed will be retrieved as well from the study database.

#### Potential effect size evaluation

In order to evaluate potential effect sizes, all effect measurements (except for motivation level) will be performed at T0, T1, and T2. The motivation level will be assessed by the lifestyle coach during each coaching session.

##### Reached lifestyle goals

The potential effect sizes of the PCFU lifestyle intervention will be determined by evaluating the percentage of CAYA cancer survivors reaching their personalized overall goals and sustaining them. After the intake sessions, the lifestyle coaches will register the personalized goal, as well as the progress made during the next sessions, in the online study database. After the last regular coaching session (T1), the coach will report whether the survivor has reached his/her lifestyle goal (yes/no). After the reflection session (T2), the coach reports whether the goal has been sustained (yes/no).

##### Physical activity

Physical activity will be assessed with the validated Short Questionnaire to Assess Health-enhancing physical activity (SQUASH) questionnaire which comprises four domains on commuting activities, physical activity at work or school, household activities, and spare time [40]. CAYA cancer survivors will be asked to indicate the number of days per week and the amount of time per day they spend on each activity. An indication of the effort for commuting and spare time activities will be asked as well. At the end, the sum score will be calculated based on the number of days per week the survivor spends at least half an hour on all the reported activities. The reliability of the SQUASH questionnaire is shown to be fairly reliable and reasonable with a Spearman correlation coefficient for overall reproducibility of 0.58 (95% CU 0.36–0.74) and a validity of 0.45 (*P* = 0.005; 95% CI 0.17–0.66) in Dutch subjects [40].

In addition to the SQUASH questionnaire, physical activity levels of participants will also be assessed objectively by an accelerometer (Yamax digiwalker SW-200®). The Yamax digiwalker SW-200® has shown to be a reliable pedometer [41]. The participants will be instructed to wear an accelerometer on their belt or waistband for 7 consecutive days from the moment they wake up until they go to sleep. The instrument responds to vertical accelerations of the hip during walking and other ambulatory movements. The accelerometer will be sent to the study participants in an envelope including clear instructions on how to wear and use it. At the end of every measurement day, the respondents will be asked to register the type and duration of physical activities performed, as well as the number of steps registered by the accelerometer for that day.

##### BMI

As part of care as usual, weight and height are measured in every survivor attending the outpatient clinic to have an indication of the survivors’ BMI. The late effect doctors will use the BMI assessment to assess the eligibility of participants for the study. After inclusion, self-reported weight measurements will be used to calculate BMI at T0, T1, and T2. Upon reporting weight at T0, T1, and T2, the survivor will be instructed to use the same scale. Height will be extracted from the medical records. To validate, self-reported weight at T0 will be compared with weight measured at the outpatient clinic.

##### Dietary intake

To assess the quality of diet, the Dutch Food Habit Questionnaire, comprising 26 questions on the type and/or frequency of consumed products, will be used [42, 43]. The questionnaire is subdivided into three categories of nutrients and food categories: (i) fat, (ii) fibres, and (iii) vegetables and fruit. All items have 2 to 4 different answer options. Scores on fat, fibres, vegetables, and fruit range from 1 to 3, where 1 corresponds to a healthy intake and 3 to an unhealthy intake. A total score (range 3–9) of the three categories can be calculated with the scoring manual.

##### Motivation level

The CAYA cancer survivor’s motivation level will be assessed during every intervention session by the coach. The coach will apply the “Stages of Change” model as developed by Prochaska and Di Clemente to assess in which of the following motivation stages the survivor is during the intervention sessions: precontemplation (“not ready”), contemplation (“getting ready”), preparation (“ready”), action, and maintenance [38]. In a study by Horiuchi et al., partial support was provided for the validity of applying the stages of change to regular exercise [44].

##### Self-efficacy

To estimate the CAYA’s cancer survivor self-efficacy level, the General Self-efficacy (GSE) scale will be used [45]. The questionnaire is comprised of items in which survivors are asked to indicate to what extent certain situations apply to them in the way they handle or act. Items are scored on a 4-point Likert-type scale, ranging from “completely true” to “completely false”. The total score is calculated by adding up the scores of the different items. The GSE scale, consisting of 10 items, has shown to have good psychometric properties with a Cronbach’s alpha of 0.85 in Dutch subjects and 0.89 in German cancer patients [46, 47]. In addition, a meta-analysis of Luszczynsksa et al. confirmed the validity of the GSE scale with correlations found with social-cognitive behaviours [47].

##### Self-management

Self-management will be assessed with the disease-specific Self-Management Screening (SeMaS) questionnaire [48]. The questionnaire, consisting of 25 items in total, screens the survivor’s capability in self-management. The items are distributed over the subscales control coordination (3 items), own effectiveness (2 items), social support (1 item), coping style (9 items), fear (4 items), depression (3 items), and skills such as computer skills (3 items). The total score can be calculated by adding up the different scores. A study by Eikelenboom et al. showed that the SeMaS is a valid tool with 31.7% of the variance explained in the Patient Activation Measure (PAM-13) instrument [49]. The psychometric characteristics of the subscales coping (problem solving) and self-efficacy were acceptable and good with a Cronbach’s alpha for internal consistency of 0.70 and 0.86, respectively [49].

### Analyses

#### Sample size

As the primary aim of this study was to assess the feasibility of the PCFU lifestyle intervention, a formal sample size calculation was not needed. Though there is no general consensus on sample sizes in feasibility studies, a pragmatic sample size of 30 participants per centre (*n*=60 in total) was chosen [50–52]. This number will allow adequate acceptability, practicality, integration/implementation, demand, and attrition testing of the PCFU lifestyle intervention. A sample size of 60 is realistic considering the number of CAYA cancer survivors invited to the survivorship care clinics per month, the study running time (20 months), and the assumption of a recruitment rate of 50%. For estimation of the potential effect sizes, a sample size of *n*=60 is also sufficient to estimate small to moderate differences in pre- and post-intervention and thus to get insight into possible effect sizes (and 95% confidence intervals) for the dichotomous primary effect measure percentage of survivors that reach the personalized lifestyle goals that were set with the coach and the continuous secondary effect measures level of physical activity, dietary intake, BMI, self-efficacy, and self-management. In case of a participant withdraws from the intervention study after inclusion and completing the T0 measurement, he/she will not be replaced by another participant. In case the participant withdraws from the study before completing the T0 measurement, he/she will be replaced by another participant when the recruitment period is still ongoing.

#### Data analyses

The feasibility of the PCFU lifestyle intervention will be determined by calculating (1) mean scores on acceptability questions in the evaluation questionnaire; (2) percentages of participants who would want chose to apply for an eHealth intervention again in the future as assessed in the evaluation questionnaire; (3) average duration for coaches needed to prepare, conduct, and report about the intervention sessions; (4) percentages of drop-outs and participants participating in all sessions; (5) percentage of eligible survivors participating in intervention; and (6) percentages of completed measurements. The exact success criteria of the PCFU lifestyle intervention study are displayed in Table 2 and will allow us to interpret the results and to determine the feasibility of the intervention and study. Other feasibility data on acceptability (usefulness and satisfaction with elements of the intervention, experiences with the coach), practicality (costs), and integration/implementation outcomes (barriers and facilitators of the intervention) will be collected to inform a future study and the replication manual, but will not be assessed with success criteria.

**Table 2:** Overview of assessments of success criteria of feasibility outcomes of the PCFU lifestyle Intervention study

Feasibility outcome	Assessment	Success criteria
Acceptability	Evaluated with a questionnaire provided at T2: 1. Rating (scale 0–10) overall intervention (survivors) 2. Rating (scale 0–10) overall intervention (coaches) 3. Rating (scale 0–10) on willingness to recommend the intervention to other survivors (survivors) 4. Rating (scale 0–10) on willingness to recommend the intervention to other co-workers or survivorship care clinics (coaches)	1. Overall rating intervention (survivors): ≥5 2. Overall score intervention (coaches): ≥5 3. Score “I would recommend the intervention to other survivors” (survivors): ≥5 4. Score “I would recommend the intervention to other co-workers/late effect clinics” (survivors): ≥5
Practicality	Evaluated with a questionnaire provided at T2: 1. Percentage of participants choosing for an eHealth intervention in the future Evaluated with the coaches’ reports: 2. Length of time needed to prepare, conduct, and report an intervention session in a database (coaches)	1. ≥50% of participants (survivors) would choose for an eHealth intervention in the future 2. Length of time needed to prepare, conduct, and report an intervention session in the database ≤90 min per session
Adherence to intervention	Evaluated with the coaches’ reports: 1. Percentage of drop-outs (survivors) 2. Percentage of participants following the sessions according to plan (survivors)	1. Percentage of drop-outs ≤10% 2. Percentage of participants following the sessions according to plan ≥80%
Demand	Evaluated with registration forms: 1. Percentage of eligible survivors who agree to participate in the PCFU Lifestyle intervention study (survivors)	1. Percentage of eligible survivors who agree to participate in the PCFU Lifestyle intervention study ≥25%
Attrition	Evaluated with the study database: 1. Percentage of complete questionnaires (survivors) 2. Percentage of complete accelerometer measurements (survivors) 3. Percentage of overall completed measurements (questionnaires and accelerometer measurements) (survivors)	1. Percentage of missing data in questionnaires ≤20% 2. Percentage of missing data in accelerometer measurements ≤20% 3. Percentage of overall completed measurements (questionnaires and accelerometer measurements) ≥75%

For exploratory testing of the potential effect sizes (and 95% confidence intervals (CI)) of the study, univariable and multivariable analyses will be performed. First, continuous data will be checked for normality and equal variances with normal probability plots and boxplots, respectively. For each of the two centres, percentages and means (± standard deviation) or medians (interquartile range) will be calculated for the baseline characteristics. The percentage of CAYA cancer survivors that improve their lifestyle on the behaviour(s) that they desire to change (yes/no) will be described at T1 and T2. Regarding the secondary outcome measures physical activity, dietary intake, BMI, self-efficacy, self-management, and motivation level, the mean (± standard deviation) or median values (interquartile range) will be calculated at T0, T1, and T2. Estimates of differences in the secondary outcomes between T0 and T1, T0 and T2, and between T1 and T2 will be explored with two-tailed paired *t*-tests and presented with 95% CIs. Subsequently, mixed models for each secondary outcome will be estimated to investigate the effect of time, centre, anti-cancer treatment factors, age at assessment, sex, socio-economic status, amount of sessions, motivational change, and baseline scores of the lifestyle behaviour variables, on the secondary outcomes. A stepwise backward selection procedure will be used to determine potential confounding factors. First, a full model containing all potential variables associated with each of the secondary outcomes will be created, after which the magnitude of associations (*β*) and 95% CIs will be carefully assessed. Variables with weak associations with the outcome measures, as well as wide 95% CIs, will be removed from the model, leaving variables with strong associations with the outcomes in the model. These are the variables that should be considered potential confounding factors for a future trial.

## Study status

The inclusion of the PCFU lifestyle intervention study started in December 2020 and was completed in July 2022. At the moment of writing, data collection is still ongoing and is planned to be finalized in May 2023. Currently, 23 participants completed the intervention and study. The results of the study are expected to be published in 2023.

## Discussion

It has been shown that the presence of lifestyle risk factors, including physical inactivity and unhealthy dietary habits, increases the already heightened risk of developing these late effects in CAYA cancer survivors. Interventions aiming at diminishing this risk by altering unhealthy lifestyle behaviours into healthy lifestyle behaviours are therefore key in the follow-up care for survivors. However, the heterogeneous character of the CAYA cancer survivor population demands a personalized approach to follow-up care and interventions. Our hypothesis is that the PCFU lifestyle intervention will positively modify lifestyle risk factors, self-efficacy, and self-management with preservation or improvement of the survivor’s health as the ultimate reward. In addition, by applying person-centred care, the PCFU lifestyle intervention can lead to improved well-being of survivors by empowering them to care for their health by adopting healthy behaviours. Evaluating the PCFU lifestyle intervention with the proposed feasibility study will help in improving the content and conduct of the lifestyle intervention in order to be implemented as part of standard care across other survivorship care outpatient clinics. In addition to that, the PCFU lifestyle intervention study has a lot of potential as it concerns an eHealth intervention for a relatively young population. Showing the feasibility of lifestyle support delivered via eHealth can clear the way for other distance-delivered eHealth interventions to be implemented.

The potential strengths of the PCFU lifestyle intervention are mostly related to the personalized character of the intervention following a consultation according to the principles of person-centred care. The content of the intervention is completely adapted to the survivor’s preferences and needs. In addition, delivering the intervention via video calling can relieve survivors from unnecessary visits to survivorship care clinics and can therefore better fit into the personal lives of survivors.

Although this seems to be a promising approach of improving lifestyle behaviours of CAYA cancer survivors, several limitations need to be considered when interpreting the results of the PCFU lifestyle study. First, online self-reported questionnaires are used. Participants may use socially desirable answers when filling in the questionnaire or may forget activities that they performed. Despite that, as almost all questionnaires used in this study are validated and widely accepted, we do not believe this to be a problem. Second, we chose to apply the “Stages of Change Model” of Prochaska and DiClemente for both intervention purposes as well as an outcome measure. However, a limitation of this model is the fact that boundaries between the “stages” are rather arbitrary selected and therefore it may be difficult for coaches to assess the appropriate stage of change in survivors during the intervention. However, as the lifestyle coaches are trained in using the “Stages of Change”, we believe they can still assess these stages properly. In addition, we believe that this feasibility study will also provide input on the usefulness of applying the “Stages of Change” as part of the intervention or as an outcome measure for the future. Third, as the GSE scale will only provide info on the survivor’s self-efficacy on a general level, we cannot make any statements on potential effect sizes of self-efficacy regarding adopting healthier behaviours. However, one of the aims of this intervention is also to empower and enhance self-efficacy in survivors in their daily lives. Therefore, we believe that the GSE scale will provide us valuable information as well. Fourth, for practical reasons, survivors are instructed to weigh themselves at home with their own weighing scale. These scales are not validated and survivors may not be honest with reporting their weight. Yet, as the survivors use the same scale for all the three measurements, the effect of the PCFU lifestyle intervention regarding weight can still be noticed. Fifth, due to time and travel burden for survivors to come to the survivorship care clinic at every measurement point, height measurements at T1 and T2 cannot be conducted by healthcare professionals. For this reason, height may therefore not be accurate for the younger participants who may potentially have grown between the T0, T1, and T2 measurements. Lastly, wearing an accelerometer may influence the physical activity patterns of the participants leading to a higher physical activity level on days where the participants wear them. In addition, it only registers vertical movements and no weight bearing exercises such as cycling and swimming.

The results of this feasibility study will be used to assess both the feasibility and potential effect sizes of the intervention to determine whether a full-scale trial is feasible and/or adaptations of the PCFU lifestyle intervention are needed in order to proceed with the intervention. In addition, the results will, together with the results of a systematic review and qualitative studies on barriers and facilitators on adopting a healthy lifestyle, inform the manual which will be written to disseminate the PCFU lifestyle intervention. By publishing the manual, the PCFU project aims to help implementation of the PCFU lifestyle intervention at other European childhood cancer survivorship care clinics.

## Supplementary Information

**Additional file 1.** SPIRIT 2013 Checklist.

**Additional file 2.** The TIDieR (Template for Intervention Description and Replication) Checklist.

**Additional file 3.** Behavioural Change Techniques and their presumed Mechanisms of Action.

## Data Availability

The PanCareFollowUp project aims to comply with all the four FAIR principles and to share individual de-identified data upon request. At the moment of writing, both the PanCareFollowUp project and the Radboud University Medical Centre are working on establishing the conditions and means to share the data. Requests for de-identified data should be made to the corresponding author (EB).

## Abbreviations

BMI: Body mass index
CAYA: Childhood, adolescent, and young adult
GSE: General Self-Efficacy
PCFU: PanCareFollowUp
RICk: Readiness, importance, confidence, knowledge
SeMaS: Self-Management Screening
SQUASH: Short Questionnaire to Assess Health-enhancing physical activity
WHO: World Health Organization
