# Supplementary material for: Person-centred online lifestyle coaching in childhood, adolescent, and young adult cancer survivors: protocol of the multicentre PanCareFollowUp lifestyle intervention feasibility study
Source: Pilot Feasibility Stud. 2022 Dec 16;8:260. doi: 10.1186/s40814-022-01221-x (PMC9756491; doi:10.1186/s40814-022-01221-x)
Supplement: Supplementary file 3 — Additional file 3. Behavioural Change Techniques and their presumed Mechanisms of Action. [file 40814_2022_1221_MOESM3_ESM.docx]

**Additional file 3. Behavioural Change Techniques and their presumed Mechanisms of Action**

**Table 2. Behaviour Change Techniques applied in the PCFU Lifestyle intervention linked with their presumed Mechanisms of Action**

| **Behaviour Change Techniques** | **Mechanisms of Action** |
| --- | --- |
| Goal setting (behaviour) | Behavioural regulation |
| Goal setting (outcome) | Goals |
| Problem solving | Beliefs about capabilities  Environmental context and resources  Skills |
| Action planning | Behavioural regulation |
| Review behavioural goals | Behavioural regulation |
| Review of outcome goals | Goals |
| Feedback on behaviour | Knowledge  Subjective norms |
| Self-monitoring of outcomes of behaviour | Behavioural regulation |
| Social support (unspecified) (including motivational interviewing) | Social influences  Social role and identity |
| Information about health consequences | Knowledge  Beliefs about consequences  Attitude towards the behaviour  Perceived susceptibility/vulnerability  Intention |
| Prompts/ cues | Memory, attention, and decision processes  Behavioural cueing  Environmental context and resources |
| Remove access to the reward | Environmental context and resources |
| Behaviour substitution | Behavioural regulation |
| Habit reversal | Behavioural regulation  Behavioural cueing  Memory, attention and decision processes |
| Non-specific reward | Reinforcement |
| Social reward | Reinforcement |
| Non-specific incentive | Motivation |
| Self-incentive | Motivation |
| Incentive outcome | Motivation |
| Self-reward | Reinforcement |
| Verbal persuasion about capability | Beliefs about capabilities |
| Focus on past success | Beliefs about capabilities |
